# Chromosome-level genome assembly of Siberian kale (*Brassica napus* subsp. *pabularia*)

**DOI:** 10.1038/s41597-026-06913-0

**Published:** 2026-02-27

**Authors:** Xi Shan, Minghao Qu, Wei Zhang, Shenyun Wang, Fangwei Yu, Meng Ni, Lei Gao, Xiaolin Yu, Jianbin Li

**Affiliations:** 1 Institute of Vegetable Crops, Jiangsu Academy of Agricultural Sciences, Nanjing, 210014 China; 2 Zhenjiang Agricultural Research Institute, Jurong, 212400 China; 3 State Key Laboratory of Plant Diversity and Specialty Crops, Wuhan Botanical Garden, Chinese Academy of Sciences, Wuhan, 430074 China; 4 Oil Crops Research Institute, Chinese Academy of Agricultural Sciences, Key Laboratory of Biology and Genetic Improvement of Oil Crops, Ministry of Agriculture and Rural Affairs, Wuhan, 430062 China; 5 Hubei Hongshan Laboratory, Wuhan, 430070 China; 6 Jiangsu Key Laboratory for Horticultural Crop Genetic Improvement, Nanjing, 210014 China; 7 Department of Horticulture, College of Agriculture and Biotechnology, Zhejiang University, Hangzhou, 310058 China; 8 Wuxi Dimode Biotechnology & Seed Industry Technology Co., Ltd., Wuxi, 214105 China

**Keywords:** Chromosomes, Genomics

## Abstract

Siberian kale (*Brassica napus* subsp. *pabularia*, AACC, 2n = 38) is a distinct subspecies of *B. napus*, characterized by its deeply lobed leaves and primarily cultivated as a nutritious leafy vegetable. Here, we present a chromosome-level genome of Beta, a Siberian kale variety, integrating Illumina short reads, PacBio HiFi long reads, and Hi-C data. The final assembly size is 1,078.8 Mb, with a scaffold N50 of 57.5 Mb and a genome BUSCO completeness of 99.7%. 954.0 Mb (88.4%) of sequences were successfully anchored to 19 pseudo-chromosomes. The configuration of Beta genome chromosomes is consistent with the distribution of ten A subgenome and nine C subgenome chromosomes in rapeseed. In total, 98,882 protein-coding genes were predicted *ab initio* in the Beta genome, with an average gene length of 1,997 bp, and 90,415 (91.44%) genes were functionally annotated. Overall, the high-quality genome provides a valuable resource for bridging current knowledge gaps and offers key genetic insights into deeply lobed leaf formation and improvement of *Brassica* crops.

## Background & Summary

Siberian kale (*Brassica napus* subsp. *pabularia*) is a currently recognized subspecies of the allotetraploid species *Brassica napus* (AACC, 2n = 38), a species that originated from hybridization between *B. rapa* (AA, 2n = 20) and *B. oleracea* (CC, 2n = 18) approximately 6,800–12,500 years ago^1–4^. Siberian kale is a leafy vegetable rich in carotenoids, fat-soluble nutrients, and glucosinolates, and is cultivated worldwide for human consumption^5^. It resembles curly-leaved kale types and exhibits superior cold-tolerance and disease resistance compared to other kale varieties^5^. The leaf shape of Siberian kale, characterized by deeply lobed leaves, differs significantly from that of most rapeseed (*B. napus* subsp. *oleifera*) and rutabaga (*B. napus* subsp. *rapifera*) varieties, which typically have toothed or entire leaf margins^6–9^. Lobed leaves are more effective at optimizing canopy structure, thereby promoting improved light penetration, air circulation, and heat dissipation, and hold great potential for promoting hybrid seed production and optimizing high-density planting, particularly in support of mechanized agriculture within *Brassica* improvement^10–12^. However, the limited availability of reference genome for Siberian kale hinders the exploration of its genomic information and genetic diversity. Therefore, sequencing and assembling the genome of Siberian kale can help bridge these knowledge gaps, facilitate comparative genomic analyses, and advance the genetic improvement of *Brassica* crops.

**Fig. 1:**
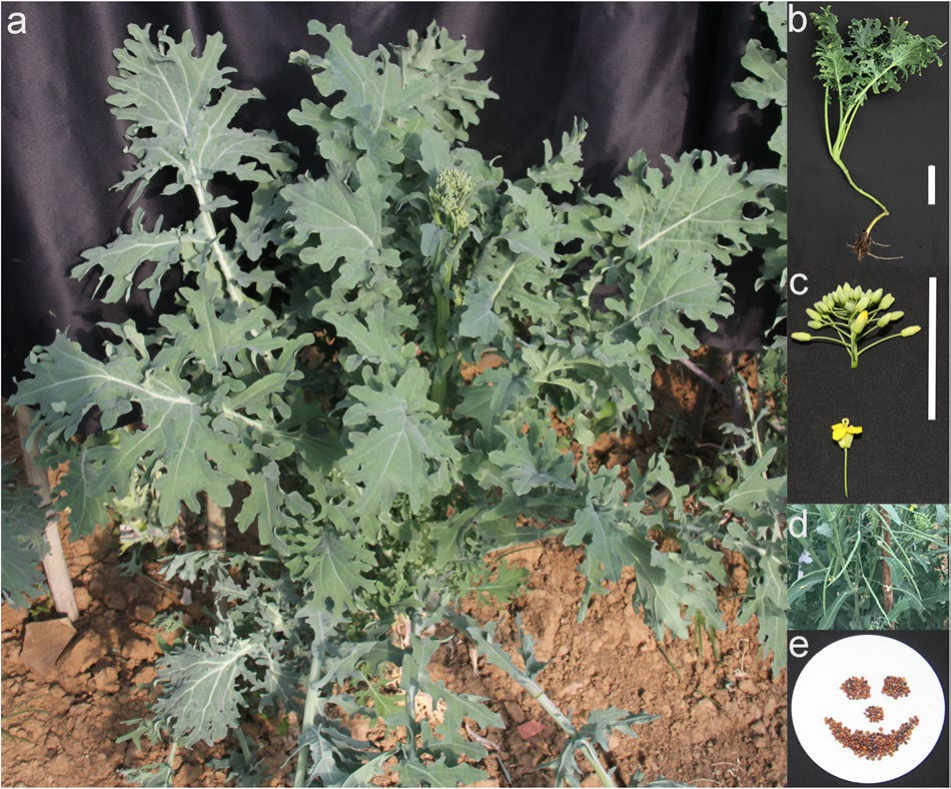
Morphological characteristics of Beta. (**a**) Bolting-stage plant; (**b**) Rosette-stage plant; (**c**) Flower bud and flower; (**d**) Siliques; (**e**) Seeds. Bar = 5 cm.

In this study, we collected a Siberian kale variety named Beta, a fast-growing leafy vegetable well-adapted to dense planting and year-round cultivation. The plants exhibit upright and vigorous growth, with gray-green, deeply lobed leaves bearing sparse surface hairs (Fig. 1a,b). They produce typical yellow cruciform flowers (Fig. 1c), followed by slender, cylindrical siliques (Fig. 1d), and finally develop nearly spherical seeds ranging in color from brown to dark brown (Fig. 1e). However, due to the lack of a high-quality reference genome for Beta, identifying candidate genes associated with key agronomic traits and breeding superior varieties remains challenging. Here, we have generated a chromosome-level genome assembly of Beta using PacBio high-fidelity (HiFi) long reads, Illumina short reads and high-throughput chromosome conformation capture (Hi-C) data first in this study. The final genome assembly totaled 1,078.8 Mb, comprising 1,870 contigs with a contig N50 of 7.1 Mb. After Hi-C scaffolding, the assembly achieved a scaffold N50 of 57.5 Mb. Up to 954.0 Mb (88.4%) of the assembled sequences were successfully anchored to 19 pseudo-chromosomes. A total of 98,882 protein-coding genes were predicted, with 90,415 (91.44%) genes functionally annotated in at least one database. This genome sequence serves as a valuable resource for exploring the molecular basis of agronomic traits in Beta and will further advance genetic improvement research.

## Methods

### Sample collection and sequencing

Fresh young leaves of the Beta variety were collected from the experimental field of the Jiangsu Academy of Agricultural Sciences in Nanjing, Jiangsu Province, China (118.88°E, 32.03°N). High molecular weight DNA of Beta was isolated from the leaves of seedling plants using the cetyltrimethylammonium bromide (CTAB) method^13^.

For Illumina short-read sequencing, Sequencing library was constructed using the Truseq Nano DNA HT Sample preparation Kit (Illumina USA) following manufacturer’s instructions. Genomic DNA was fragmented by sonication to a size of 350 bp DNA fragments^14,15^. After end polishing, A-tailing, and ligation with full-length adapters, PCR amplification was performed. The resulting PCR products were purified using the AMPure XP system. Library size distribution was assessed using the Agilent 2100 Bioanalyzer^16^. The constructed library was then sequenced on the Illumina HiSeq X Ten platform, generating a total of 104.84 Gb paired-end 150 bp reads, with a coverage depth of approximately 94.75 × of the genome (Table 1).

**Table 1:** Summary of sequencing data for Beta.

Library type	Raw data (Gb)	Clean data (Gb)	Read length (bp)	Coverage (×)
Illumina	104.84	102.21	150	94.75
PacBio HiFi	645.26 (Subreads)	40.87 (HiFi reads)	14,813 (N50)	37.88
Hi-C	211.36	204.62	150	189.67
RNA	13.73	13.63	150	

For PacBio HIFI sequencing, genomic DNA was fragmented to ~15 Kb fragments by g-TUBE (Covaris, Inc., MA, USA) for HiFi library construction. The HiFi library was prepared using the SMRTbell Express Template Prep Kit 2.0 and sequenced on the PacBio Sequel IIe platform (Pacific Biosciences, Menlo Park, USA) in Circular Consensus Sequencing (CCS) mode. A total of 40.87 Gb HiFi reads were obtained, with a read N50 size of 14,813 bp, corresponding to approximately 37.88 × genome coverage (Table 1).

For Hi-C sequencing, chromatin was performed by 4% formaldehyde and digested using the *DpnII* (NEB) restriction endonuclease^17^. Biotin-14-dCTP was incorporated during end-repair, followed by proximity and fragmentation into 300–700 bp using sonication. Biotin-labeled DNA fragments were enriched using streptavidin beads for library construction. The resulting library was sequenced and a total of 211.36 Gb (189.67×) Hi-C data was acquired (Table 1).

For genome annotation, RNA-seq was performed on leaves and flowers of Beta. The two libraries were prepared using NEBNext UltraTM RNA Library Prep Kit (NEB, USA) following the manufacturer’s instructions and sequenced on the Illumina NovaSeq 6000 platform. A total of 13.73 Gb RNA-seq reads were obtained for Beta (Table 1).

### Karyotype analysis

**Fig. 2:**
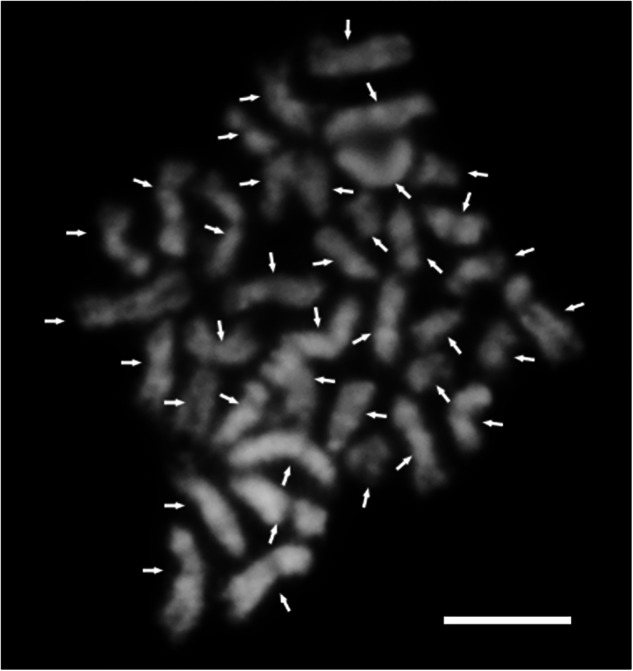
Karyotype analysis of Beta. Chromosomes are indicated by white arrows. Bar = 5 µm.

Root tips (2–3 cm in length) from Beta were excised and placed in a 0.5 mL Eppendorf tube pre-moistened with ddH₂O. The tube was then transferred to a gas chamber, filled with nitrous oxide (N₂O) to a pressure of 0.9–1.0 MPa, and incubated for 2 hours. Following this, 90% pre-cooled glacial acetic acid was added to the tube, and the sample was kept on ice for 10 minutes. After two washes with ddH₂O, the white region of the root tip was excised and placed in 25 μL of enzyme solution consisting of cellulase and pectinase (in a 3:1 ratio) for enzymatic digestion at 37 °C for 1 hour. After digestion, the root tips were washed three times with 70% alcohol. Dissecting needles were then used to thoroughly crush the root tips in the remaining alcohol, followed by centrifugation at 4000 rpm to pellet the cells. Depending on the number of root tips, 25–45 μL of ice-cold acetic acid was added. An 8 μL aliquot of the cell suspension was dropped onto a slide, which was allowed to dry at 23 °C before being used for chromosome examination. Karyotype analysis showed that Beta possesses 38 chromosomes (2n = 38) (Fig. 2).

### Genome survey and assembly

**Fig. 3:**
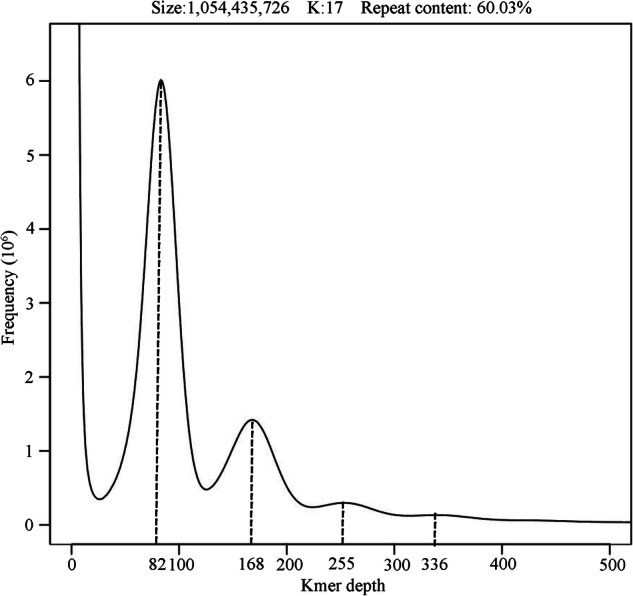
Frequency distribution of Beta using k-mer analysis. The x-axis represents the k-mer depth and y-axis represents the frequency of k-mer corresponding to the depth.

A genome survey was performed using Illumina short reads through k-mer analysis. The raw PE150 reads were trimmed adapter and low-quality reads by fastp v0.21.0 with default parameters^18^. PCR duplicates were removed using FastUniq v1.1^19^. The genome size was then estimated based on 17-mer frequency with Jellyfish v2.1.4^20^ and GenomeScope v2.0^21^. The k-mer analysis revealed four peaks with depths of 82, 168, 255, and 336, forming a clear 1:2:3:4 ratio, and the presence of a secondary peak exceeding the homozygous peak; together, these features support an allotetraploid genome architecture (Fig. 3). The genome size was estimated to be 1054.4 Mb, and the repeat content was estimated to be approximately 60.03% (Fig. 3).

**Fig. 4:**
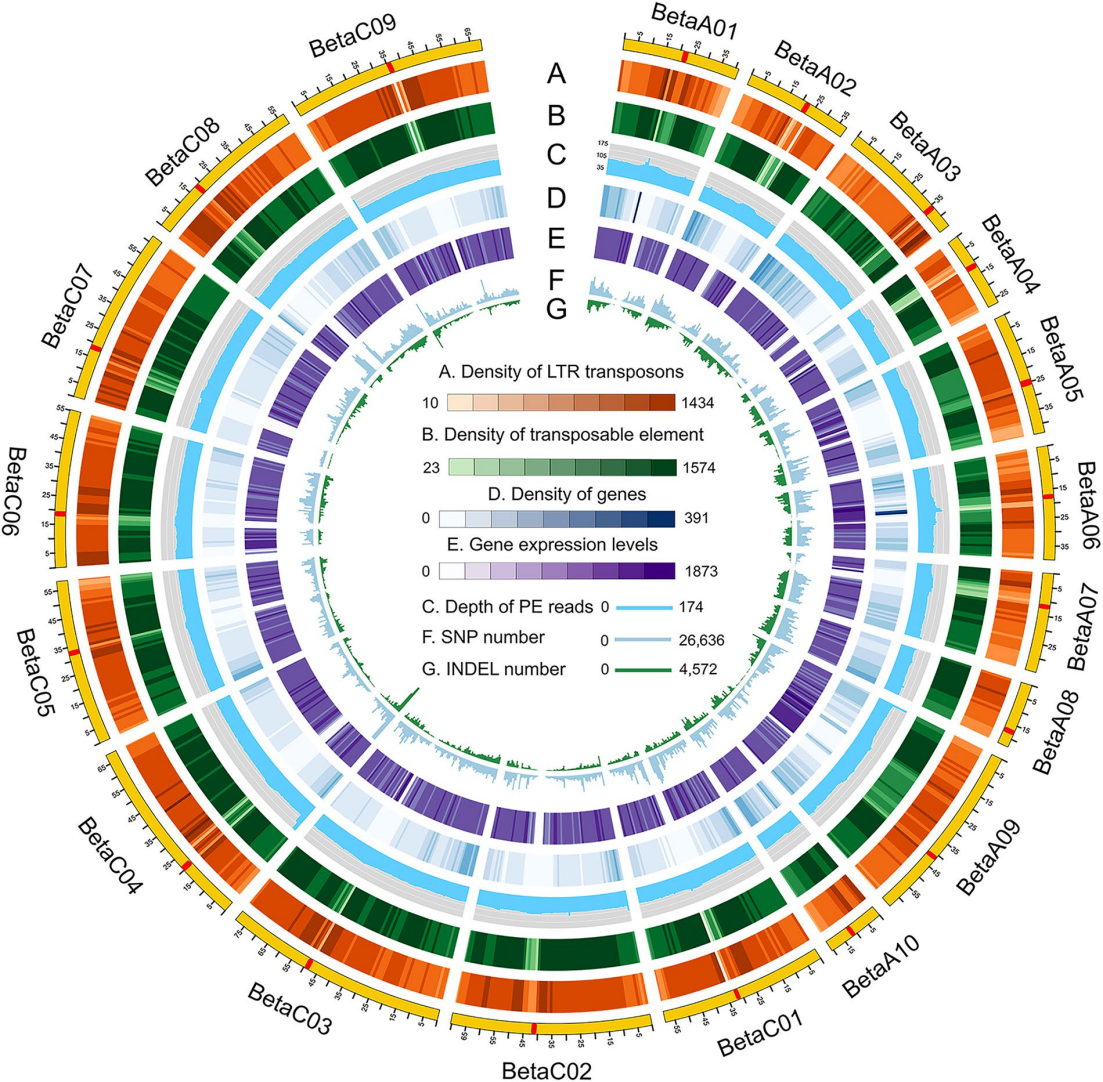
Circos plot of 19 chromosomes of Beta genome. The data from outside to inside are as follows: (A) density of LTR transposons, (B) density of transposable element, (C) depth of PE reads, (D) density of genes, (E) gene expression levels, (F) SNP number, (G) INDEL number. The centromeric regions are marked with red boxes in the outermost circle. Windows size is 500 kb.

**Fig. 5:**
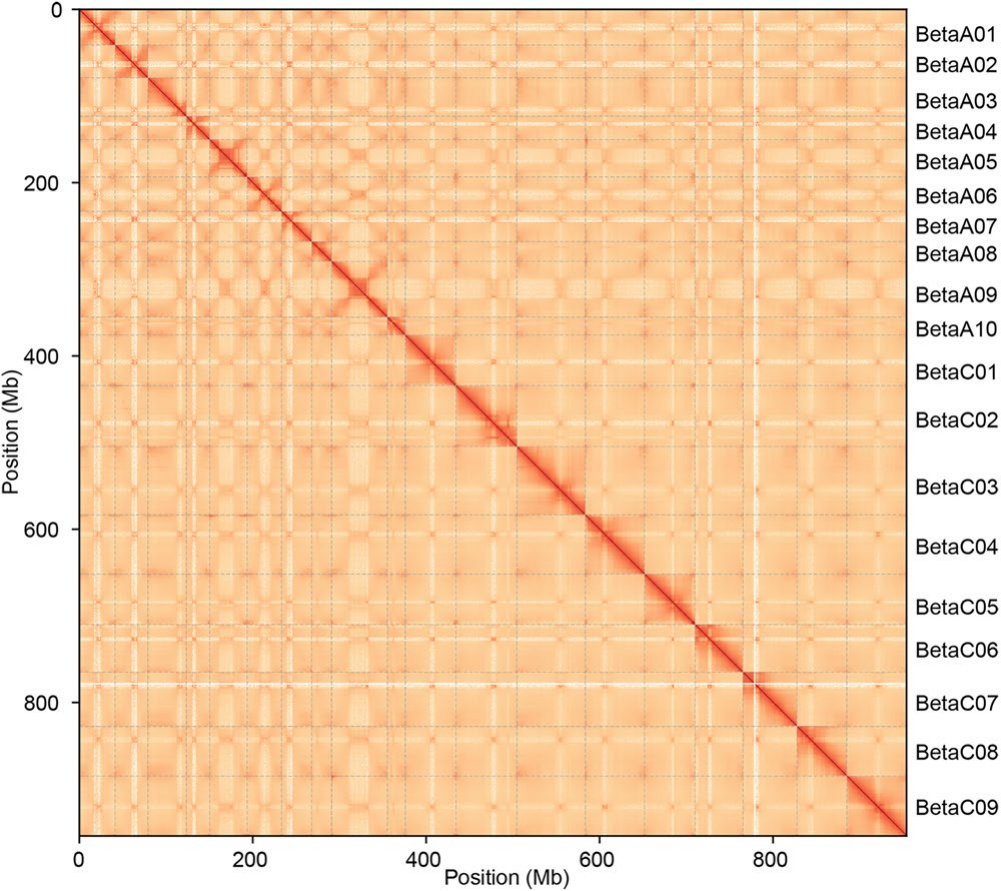
Hi-C interaction heatmap of the Beta genome at the chromosome level.

*De novo* assembly of PacBio HiFi reads was performed using hifiasm v0.16^22^ with default parameters. The final genome assembly had a cumulative size of 1,078.8 Mb, comprising 1,870 contigs with a contig N50 of 7.1 Mb (Table 2). The clean Hi-C reads were aligned to the contig-level genome by Juicer v1.9.9^23^. The primary contigs were anchored into chromosomes using 3D-DNA v180419^24^. Assembly errors were manually inspected and corrected using Juicebox v1.13^25^. Finally, the assembly achieved a scaffold N50 of 57.5 Mb (Table 2). The resulting chromosome-level genome for Beta was 954.0 Mb, with 88.42% of sequences anchored to 19 pseudochromosomes (Table 3; Fig. 4). The lengths of A and C subgenomes were 375.9 Mb and 578.1 Mb, respectively (Table 3; Fig. 4). The Hi-C interaction heatmap showed these 19 pseudo-chromosomes were well linked along the diagonal line, indicating high quality of chromosome construction (Fig. 5).

**Table 2:** Assessment of genome assembly in Beta.

Feature	Beta
Assembly length (bp)	1,078,777,375
Pseudochromosome length (bp)	953,967,409
Longest contig (bp)	26,347,464
Contig number	1,870
Contig N50 (bp)	7,054,300
Contig L50	45
Scaffold N50 (bp)	57,461,123
Merqury (QV)	47.77
K-mer completeness (%)	98.76
Anchor rate (%)	88.42
Mapping rate (%)	98.83
BUSCO completeness (%)	99.70

**Table 3:** Summary of pseudochromosome lengths in Beta genome.

Subgenomes	Chromosomes	Length (bp)
A subgenome	A01	41,159,364
	A02	37,885,386
	A03	44,327,209
	A04	26,917,064
	A05	43,243,519
	A06	39,743,173
	A07	34,829,559
	A08	22,890,646
	A09	64,313,872
	A10	20,568,429
C subgenome	C01	58,398,963
	C02	70,379,099
	C03	78,808,587
	C04	68,268,749
	C05	58,290,432
	C06	55,219,305
	C07	62,349,480
	C08	57,461,123
	C09	68,913,450
Pseudochromosome		953,967,409
Total assembly		1,078,777,375

### Genome annotation

The repeat libraries were combined from two *de novo* prediction strategies. RepeatModeler v2.0.1^26^ was used with the RMBLAST engine to construct a repeat library from the Beta genome. The RepeatScout^27^ and RECON^28^ modules within RepeatModeler were used to identify non-LTR library. The long terminal repeat (LTR) libraries were obtained by LTR_harvest^29^ and LTR_FINDER v1.07^30^ with default parameters. The LTR_retriever-2.9.0^31^ merged these two LTR screen output files into a LTR library. RepeatMasker-4.1.2^32^ was used to integrate the results above to generate the final repetitive annotation. The results indicated that the genome exhibits a high level of repetitiveness, with repetitive sequences accounting for 58.45% of the total genome (Table 4). The proportion of retroelements (30.88%) was higher than that of DNA transposons (9.01%). Among the transposable elements, long terminal repeat (LTR) retrotransposons were the most abundant, constituting 28.01% of the genome (Table 4), with the highest LTR density observed near centromeric regions (Fig. 4).

**Table 4:** Summary of transposable elements in Beta genome.

Repeat elements			Number	Length (bp)	Percentage (%)
Class I: Retroelements			327,166	333,114,041	30.88
	SINEs		1,907	425,850	0.04
	LINEs		35,731	30,549,206	2.83
		LINEs/L2/CR1/Rex	3,073	715,320	0.07
		LINEs/L1/CIN4	31,332	29,253,856	2.71
	LTR elements		289,528	302,138,985	28.01
		BEL/Pao	296	228,070	0.02
		Ty1/Copia	80,132	94,821,115	8.79
		Gypsy/DIRS1	105,656	146,267,648	13.56
		Retroviral	350	72,815	0.01
Class II: DNA transposons			164,765	97,203,265	9.01
	hobo-Activator		47,550	16,210,181	1.50
	Tc1-IS630-Pogo		23,026	5,369,981	0.50
	Tourist/Harbinger		19,801	8,095,671	0.75
Rolling-circles			13,865	11,872,301	1.10
Unclassified			292,848	112,279,560	10.41
Satellites			3,958	75,921,449	7.04
Simple repeats			1,616	175,159	0.02
Total				630,565,775	58.45

For the prediction of protein-coding genes, the combination of RNA-seq were filtered by fastp v0.21.0^18^. All clean RNA-seq reads were mapped to the Beta genome by HISAT2 v2.2.1^33^. StringTie2^34^ was used to extract the alignment information and TransDecoder v5.5.0 (https://github.com/TransDecoder/TransDecoder) was used to predict these results as protein coding regions. The open reading frames (ORFs) predicted by TransDecoder were further served as a training reference for SNAP^35^, GlimmerHMM and AUGUSTUS^36^, which obtained the *ab initio* predictions. In total, 98,882 protein-coding genes were predicted *ab initio* in the Beta genome, with an average gene length of 1,997 bp and an average CDS length of 1,002 bp (Table 5). The predicted protein-coding genes of Beta were assessed for BUSCO completeness using BUSCO v5.3.2 (Benchmarking Universal Single-Copy Orthologs) with the embryophyta_odb10 database, resulting in a score of 95.1%. Among these, 77.60% of conserved single-copy genes and 13.80% of duplicated genes were detected in the A subgenome, while 78.40% of conserved single-copy genes and 12.90% of duplicated genes were identified in the C subgenome (Table 5).

**Table 5:** Characteristics and BUSCO completeness of the protein-coding genes in Beta.

	Beta
Gene number	98,882
Average gene length (bp)	1,997
Average CDS length (bp)	1,002
Complete BUSCOs	95.10%
Single-copy BUSCOs	9.40%
Duplicated BUSCOs	85.70%
BUSCOs of A subgenome	91.40%
Single-copy BUSCOs	77.60%
Duplicated BUSCOs	13.80%
BUSCOs of C subgenome	91.30%
Single-copy BUSCOs	78.40%
Duplicated BUSCOs	12.90%

**Note:** BUSCO values were calculated based on the predicted protein-coding genes.

For the functional annotation of the protein-coding genes, the protein sequences of *Brassica rapa*^37^, *B. oleracea*^38^, *B. napus*^39,40^, *B. juncea*^41^, *Arabidopsis thaliana*^42^ and UniProt-Swiss-Prot database were taken as homologous utilization. All lines of evidence (RNA-seq, *ab initio*, and homology) were integrated using MAKER v3.01.04^43^ with default parameters to predict a consensus region of protein coding genes. The genes predicted above were aligned to five public databases to be functionally annotated, including GenBank nr, Uniprot, Interpro, GO and KEGG, by using DIAMOND v2.0.13^44^. As a result, a total of 98,616 genes (91.44% of the predicted protein-coding genes) obtained functional annotations in at least one database (Table 6).

**Table 6:** Summary of functional annotations of protein-coding genes.

Database	Annotated number	Percentage (%)
NR	90,348	91.37
Swiss-Prot	58,411	59.07
KEGG	84,820	85.78
GO	49,174	49.73
TrEMBL	90,006	91.02
InterPro	66,082	66.83
Total	90,415	91.44

#### Synteny analysis between the Beta and ZS11 (rapeseed) genomes

**Fig. 6:**
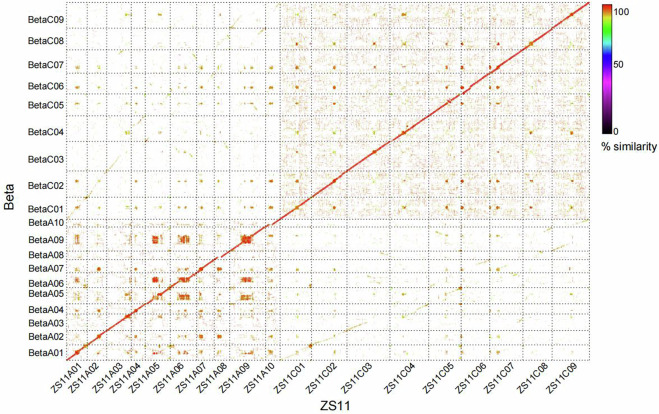
Synteny analysis between the Beta and ZS11 genomes. The colors in the figure indicate the sequence similarity.

Pairwise alignments between the Beta genome and the published rapeseed ZS11 genome were performed using MUMMER4 software^45^. Collinear regions were extracted from the alignment results using syri-1.4 software^46^. The results showed that the Beta genome had the highest proportion of collinear regions with the A subgenome and C subgenome of ZS11, respectively (Fig. 6).

## Data Records

The raw sequencing data have been deposited in the National Center for Biotechnology Information (NCBI) Sequence Read Archive (SRA) under the accession number SRP557759^47^ and in the Genome Sequence Archive (GSA) of the National Genomics Data Center (NGDC) under the accession number CRA022141^48^. The dataset includes Illumina short reads (SRR32020465 from SRA, CRR1540294 from GSA), PacBio HiFi reads (SRR32020466, CRR1540293), Hi-C data (SRR32020464, CRR1540295), and RNA-seq reads (SRR32020463, SRR32020462, CRR1540296, CRR1540297). The genome assembly and annotation data has been deposited in the GenBank database under accession number JBTLXE000000000^49^ and is also publicly available on Figshare^50^, respectively.

## Technical Validation

BUSCO 5.3.2 was used to assess the completeness of the Beta genome assembly^51^, yielding a completeness score of 99.70%. Merqury v2020-01-29^52^, a k-mer-based assessment tool, was used to evaluate the genome assembly, yielding a k-mer completeness of 98.76% and a consensus quality value (QV) of 47.77. Illumina short reads were aligned to the genome assembly using BWA v0.7.17^53^, with a mapping rate of 98.83%. LTR_harvest and LTR_retriever were used to evaluate the LTR assembly index (LAI), producing a score of 18.77^54^.

To further assess large-scale structural accuracy, synteny analysis was performed between the Beta genome and the rapeseed cultivar ZS11. Approximately 85.52% of the Beta genome exhibited collinearity with ZS11, with aligned regions having an average sequence identity of 92.01%, revealing a high degree of structural conservation. These results provide robust evidence for the accuracy and contiguity of the chromosome-scale assembly of the Beta genome and support its suitability for downstream comparative genomics, gene discovery, and cross-lineage trait mapping. Overall, these evaluations confirm that the Beta genome assembly is highly complete, contiguous, and accurate.

## Data Availability

All sequencing data generated in this study are available in the NCBI SRA under the accession number SRP557759: https://identifiers.org/ncbi/insdc.sra:SRP557759. The raw sequencing data has been uploaded to the GSA under the accession number. CRA022141: https://ngdc.cncb.ac.cn/gsa/browse/CRA022141. The genome assembly and annotation data have been deposited in the GenBank database under the accession number JBTLXE000000000: https://identifiers.org/ncbi/insdc:JBTLXE000000000. The genome assembly and annotation data are also available in the figshare: 10.6084/m9.figshare.28191848.

## Code availability

No custom scripts or codes were used during this study. The versions of software and parameters used have been referenced in the Methods section. Procedures not explicitly described in the Methods were performed using default parameters, unless otherwise specified.

## References

[1] Chalhoub, B. *et al*. Early allopolyploid evolution in the post-Neolithic *Brassica napus* oilseed genome. *Science* **345**, 950–953 (2014). 25146293 10.1126/science.1253435

[2] Edwards, D., Batley, J., Parkin, I. & Kole, C. (eds.). Genetics, genomics and breeding of oilseed *Brassicas*. (CRC Press, 2011).

[3] Lu, K., Wei, L., Li, X., Wang, Y. & Li, J. Whole-genome resequencing reveals *Brassica napus* origin and genetic loci involved in its improvement. *Nat Commun* **10**, 1154 (2019). 30858362 10.1038/s41467-019-09134-9 PMC6411957

[4] Kimber, D. S. & McGregor, D. I (eds.). Brassica oilseeds: production and utilization. (National Institute of Agricultural Botany, Cambridge, 1995).

[5] Parry, F. A., Dar, W. A., Bhat, B. A. & Sultan, T. Response of different combinations of organic manures and bio-fertilizers on growth and yield of kale Cv. Gurez local (Siberian kale) under high altitude climatic conditions of Gurez-J&K. *J Pharmacogn Phytochem* **10**, 155–157 (2021).

[6] Gazave, E. *et al*. Population genomic analysis reveals differential evolutionary histories and patterns of diversity across subgenomes and subpopulations of *Brassica napus* L. *Front Plant Sci* **7**, 525 (2016). 27148342 10.3389/fpls.2016.00525 PMC4838616

[7] Havlickova, L. *et al*. Validation of an updated associative transcriptomics platform for the polyploid crop species *Brassica napus* by dissection of the genetic architecture of erucic acid and tocopherol isoform variation in seeds. *Plant J* **93**, 181–192 (2017). 29124814 10.1111/tpj.13767 PMC5767744

[8] An, H. *et al*. Transcriptome and organellar sequencing highlights the complex origin and diversification of allotetraploid *Brassica napus*. *Nat Commun* **10**, 2878 (2019). 31253789 10.1038/s41467-019-10757-1 PMC6599199

[9] Wen, Y. *et al*. Genetics and agronomic traits of 2 mutants with lobbed-leaf margins in *Brassica napus* L. *Chin J Oil Crop Sci* **39**, 13–17 (2017).

[10] Vogel, S. Leaves in the lowest and highest winds: temperature, force and shape. *New Phytol* **183**, 13–26 (2009). 19413689 10.1111/j.1469-8137.2009.02854.x

[11] Tu, Y. *et al*. Character and genetic analysis of lobed-leaf traits in *Brassica napus*. *Chin J Oil Crop Sci* **35**, 93–96 (2013).

[12] Zhu, Q. H. *et al*. Integrated mapping and characterization of the gene underlying the okra leaf trait in *Gossypium hirsutum* L. *J Exp Bot* **67**, 763–774 (2016). 26567355 10.1093/jxb/erv494 PMC4737076

[13] Almakarem, A. S. A., Heilman, K. L., Conger, H. L., Shtarkman, Y. M. & Affiliations, S. O. R. Extraction of DNA from plant and fungus tissues *in situ*. *BMC Res Notes* **6**, 266 (2012). 10.1186/1756-0500-5-266 PMC3505157 22672795

[14] An, Y. *et al*. DNA methylation analysis explores the molecular basis of plasma cell-free DNA fragmentation. *Nat Commun* **14**, 287 (2023). 36653380 10.1038/s41467-023-35959-6 PMC9849216

[15] Lakha, W. *et al*. DNA fragmentation and quality control analysis using Diagenode shearing systems and Fragment Analyzer. *Nat methods* **13**, iii–iv (2016).

[16] Robin, J. D., Ludlow, A. T., LaRanger, R., Wright, W. E. & Shay, J. W. Comparison of DNA quantification methods for next generation sequencing. *Sci Rep* **6**, 24067 (2016). 27048884 10.1038/srep24067 PMC4822169

[17] Louwers, M., Splinter, E., Driel, R., Laat, W. & Stam, M. Studying physical chromatin interactions in plants using Chromosome Conformation Capture (3C). *Nat Protoc* **4**, 1216–1229 (2009). 19644461 10.1038/nprot.2009.113

[18] Chen, S., Zhou, Y., Chen, Y. & Gu, J. Fastp: an ultra-fast all-in-one FASTQ preprocessor. *Bioinformatics* **34**, i884–i890 (2018). 30423086 10.1093/bioinformatics/bty560 PMC6129281

[19] Xu, H. *et al*. FastUniq: A Fast De Novo Duplicates Removal Tool for Paired Short Reads. *PLoS One* **7**, e52249 (2012). 23284954 10.1371/journal.pone.0052249 PMC3527383

[20] Marçais, G. & Kingsford, C. A fast, lock-free approach for efficient parallel counting of occurrences of k-mers. *Bioinformatics* **27**, 764–770 (2011). 21217122 10.1093/bioinformatics/btr011 PMC3051319

[21] Vurture, G. W. *et al*. GenomeScope: fast reference-free genome profiling from short reads. *Bioinformatics* **33**, 2202–2204 (2017). 28369201 10.1093/bioinformatics/btx153 PMC5870704

[22] Cheng, H., Concepcion, G. T., Feng, X., Zhang, H. & Li, H. Haplotype-resolved de novo assembly using phased assembly graphs with hifiasm. *Nat Methods* **18**, 170–175 (2021). 33526886 10.1038/s41592-020-01056-5 PMC7961889

[23] Durand, N. C. *et al*. Juicebox provides a visualization system for Hi-C contact maps with unlimited zoom. *Cell Syst* **3**, 99–101 (2016). 27467250 10.1016/j.cels.2015.07.012 PMC5596920

[24] Olga, D. *et al*. De novo assembly of the *Aedes aegypti* genome using Hi-C yields chromosome-length scaffolds. *Science* **356**, 92–95 (2017). 28336562 10.1126/science.aal3327 PMC5635820

[25] Robinson, J. T. *et al*. Juicebox.js Provides a Cloud-Based Visualization System for Hi-C Data. *Cell Syst* **6**, 256–258 (2018). 29428417 10.1016/j.cels.2018.01.001 PMC6047755

[26] Flynn, J. M. *et al*. RepeatModeler2 for automated genomic discovery of transposable element families. *Proc Natl Acad Sci USA* **117**, 9451–9457 (2020). 32300014 10.1073/pnas.1921046117 PMC7196820

[27] Price, A. L., Jones, N. C. & Pevzner, P. A. De novo identification of repeat families in large genomes. *Bioinformatics* **21**, i351–i358 (2005). 15961478 10.1093/bioinformatics/bti1018

[28] Bao, Z. & Eddy, S. R. Automated de novo identification of repeat sequence families in sequenced genomes. *Genome Res* **12**, 1269–1276 (2002). 12176934 10.1101/gr.88502 PMC186642

[29] Ellinghaus, D., Kurtz, S. & Willhoef, U. LTRharvest, an efficient and flexible software for de novo detection of LTR retrotransposons. *BMC Bioinform* **9**, 18 (2008). 10.1186/1471-2105-9-18 PMC2253517 18194517

[30] Xu, Z. & Wang, H. LTR-FINDER: an efficient tool for the prediction of full-length LTR retrotransposons. *Nucleic Acids Res* **35**, 265–268 (2007). 10.1093/nar/gkm286 PMC1933203 17485477

[31] Ou, S. & Jiang, N. LTR_retriever: A highly accurate and sensitive program for identification of long terminal repeat retrotransposons. *Plant Physiol* **176**, 1410–1422 (2018). 29233850 10.1104/pp.17.01310 PMC5813529

[32] Tarailo-Graovac, M. & Chen, N. Using RepeatMasker to identify repetitive elements in genomic sequences. *Curr Protoc Bioinform* **25** (2009). 10.1002/0471250953.bi0410s25 19274634

[33] Kim, D., Paggi, J. M., Park, C., Bennett, C. & Salzberg, S. Graph-based genome alignment and genotyping with HISAT2 and HISAT-genotype. *Nat Biotechnol* **37**, 907–915 (2019). 31375807 10.1038/s41587-019-0201-4 PMC7605509

[34] Mihaela, P. *et al*. StringTie enables improved reconstruction of a transcriptome from RNA-seq reads. *Nat Biotechnol* **33**, 290–295 (2015). 25690850 10.1038/nbt.3122 PMC4643835

[35] Korf, I. Gene finding in novel genomes. *BMC Bioinform* **5**, 59 (2004). 10.1186/1471-2105-5-59 PMC421630 15144565

[36] Stanke, M., Diekhans, M., Baertsch, R. & Haussler, D. Using native and syntenically mapped cDNA alignments to improve de novo gene finding. *Bioinformatics* **24**, 637–644 (2008). 18218656 10.1093/bioinformatics/btn013

[37] Wang, X. *et al*. The genome of the mesopolyploid crop species *Brassica rapa*. *Nature Genetics* **43**, 1035–1039 (2011). 21873998 10.1038/ng.919

[38] Liu, S. *et al*. The *Brassica oleracea* genome reveals the asymmetrical evolution of polyploid genomes. *Nat Commun* **5**, 3930 (2014). 24852848 10.1038/ncomms4930 PMC4279128

[39] Song, J. M. *et al*. Eight high-quality genomes reveal pan-genome architecture and ecotype differentiation of *Brassica napus*. *Nature Plants* **6**, 34–45 (2020). 31932676 10.1038/s41477-019-0577-7 PMC6965005

[40] Schmutzer, T. *et al*. Species-wide genome sequence and nucleotide polymorphisms from the model allopolyploid plant *Brassica napus*. *Sci Data* **2**, 150072 (2015). 26647166 10.1038/sdata.2015.72 PMC4672681

[41] Yang, J. *et al*. The genome sequence of allopolyploid *Brassica juncea* and analysis of differential homoeolog gene expression influencing selection. *Nat Genet* **48**, 1225–1232 (2016). 27595476 10.1038/ng.3657

[42] Michael, T. P. *et al*. High contiguity *Arabidopsis thaliana* genome assembly with a single nanopore flow cell. *Nat Commun* **9**, 541 (2018). 29416032 10.1038/s41467-018-03016-2 PMC5803254

[43] Cantarel, B. L. *et al*. MAKER: An easy-to-use annotation pipeline designed for emerging model organism genomes. *Genome Res* **18**, 188–196 (2008). 18025269 10.1101/gr.6743907 PMC2134774

[44] Buchfnk, B., Xie, C. & Huson, D. H. Fast and sensitive protein alignment using DIAMOND. *Nat Methods* **12**, 59–60 (2014). 25402007 10.1038/nmeth.3176

[45] Marcais, G. *et al*. MUMmer4: A fast and versatile genome alignment system. *PLoS Comput Biol* **14**, e1005944 (2018). 29373581 10.1371/journal.pcbi.1005944 PMC5802927

[46] Goel, M., Sun, H., Jiao, W. B. & Schneeberger, K. SyRI: finding genomic rearrangements and local sequence differences from whole-genome assemblies. *Genome Biol.* **20**, 1–13 (2019). 31842948 10.1186/s13059-019-1911-0 PMC6913012

[47] *NCBI Sequence Read Archive.* https://identifiers.org/ncbi/insdc.sra:SRP557759 (2025).

[48] *NGDC Genome Sequence Archive.* https://ngdc.cncb.ac.cn/gsa/browse/CRA022141 (2025).

[49] *NCBI GenBank.* https://identifiers.org/ncbi/insdc:JBTLXE000000000 (2025).

[50] Qu, M. The genome assembly of Siberian kale (*Brassica napus* subsp. *pabularia*). *Figshare.*10.6084/m9.figshare.28191848 (2025). 10.1038/s41597-026-06913-0 PMC13066399 41760683

[51] Simão, F. A., Waterhouse, R. M., Ioannidis, P., Kriventseva, E. V. & Zdobnov, E. M. Busco: assessing genome assembly and annotation completeness with single-copy orthologs. *Bioinformatics* **31**, 3210–3212 (2015). 26059717 10.1093/bioinformatics/btv351

[52] Rhie, A., Walenz, B. P., Koren, S. & Phillippy, A. M. Merqury: reference-free quality, completeness, and phasing assessment for genome assemblies. *Genome Biol* **21**, 245 (2020). 32928274 10.1186/s13059-020-02134-9 PMC7488777

[53] Li, H. & Durbin, R. Fast and accurate short read alignment with Burrows–Wheeler transform. *Bioinformatics* **25**, 1754–1760 (2009). 19451168 10.1093/bioinformatics/btp324 PMC2705234

[54] Ou, S., Chen, J. & Jiang, N. Assessing genome assembly quality using the LTR Assembly Index (LAI). *Nucleic Acids Res* **46**, e126–e126 (2018). 30107434 10.1093/nar/gky730 PMC6265445

